# Expression of c-myc and PCNA in Epstein-Barr virus-associated gastric carcinoma

**DOI:** 10.3892/etm.2013.972

**Published:** 2013-02-22

**Authors:** SHIGUANG ZHU, PING SUN, YINGXIN ZHANG, LIPING YAN, BING LUO

**Affiliations:** 1 Department of Oncology, Yantai Yuhuangding Hospital, Yantai, Shandong 264000;; 2 Central Laboratory, The People’s Hospital of Liaocheng City, Liaocheng 252000;; 3 Clinical Laboratory, Qingdao Central Hospital, Qingdao 266042;; 4 Department of Microbiology, Medical College of Qingdao University, Qingdao 266021, P.R. China

**Keywords:** stomach neoplasm, Epstein-Barr virus, c-myc, proliferating cell nuclear antigen, reverse transcription-polymerase chain reaction, immunohistochemistry, progressive genes

## Abstract

The aim of this study was to detect the expression of proliferatng cell nuclear antigen (PCNA) and c-myc in Epstein-Barr virus (EBV)-associated gastric carcinoma (EBVaGC) and EBV-negative gastric carcinoma (EBVnGC), as well as the expression of EBV-encoded proteins in EBVaGC and their effect on carcinogenesis and the development of gastric cancer. The PCNA and c-myc protein levels were assessed by immunohistochemistry in 13 EBVaGC and 45 EBVnGC specimens. The expression of related genes of EBV, including EB nuclear antigen (NA)-1 and EBNA2 genes, latent membrane protein 1 (LMP1) and early genes BARF1 and BHRF1 were tested by reverse transcription-polymerase chain reaction (RT-PCR) and southern blotting. The PCNA labeling index (LI) of EBVaGCs, EBVnGCs and the corresponding adjacent tissues of EBVaGCs were 49.3768±12.1832, 14.839±7.1847, 35.613±8.3831 and 24.2735±10.1332, respectively. The PCNA LI was significantly different between EBVaGC and EBVnGC of EBVaGC (t=4.686, P<0.01). The difference between EBVaGC and corresponding adjacent tissues of EBVaGC was also significant (t=8.805, P<0.01). The expression of c-myc protein was detected in 33 of 58 (55.39%) gastric carcinomas and in 21 of 58 (36.21%) adjacent tissues. There was a significant difference between the two groups (χ^2^=4.989, P<0.05). The expression of the c-myc protein was detected in 8 of 13 (61.54%) EBVaGCs and in 25 of 45 (55.56%) EBVnGCs. The difference between the two groups was not significant (χ^2^=0.147, P>0.05). EBNA1 mRNA was detected in all 13 EBVaGC cases, while EBNA2 and LMP1 mRNA was not detected in these cases. Of the 13 EBV-positive samples, 6 exhibited BARF1 transcripts and 2 exhibited BHRF1 transcripts. c-myc expression did not correlate with the presence of EBV in EBVaGC. EBV infection may induce PCNA expression. The lack of EBNA2 and LMP1 protein expression in EBVaGC suggests that EBNA2 and LMP1 do not correlate with cell apoptosis and c-myc expression. Early genes BARF1 and BHRF1 may play an important role in the development and progression of gastric carcinomas by immortalizing epithelial cells.

## Introduction

The Epstein-Barr virus (EBV) was the first described onco-virus. Previously, research mainly focused on the role of Burkitt lymphoma, nasopharyngeal carcinoma, Hodgkin’s disease and B lymphoma in patients with acquired immuno-deficiency syndrome (AIDS) (1). It has been identified that 7–16% gastric cancer cases are EBV-positive (2,3). Therefore, the role of EBV in gastric cancer is becoming increasingly acknowledged. In this study, using immunohistochemistry, we examined the expression of PCNA and c-myc in 13 cases of EBV-associated gastric carcinoma (EBVaGC) and 45 cases of EBV-negative gastric carcinoma (EBVnGC) as the control. Additionally, the pericancer tissues were also examined. The Epstein-Barr nuclear antigen (EBNA)-1, EBNA-2 and latent membrane protein 1 (LMP1) genes, which are expressed in the virus, were assayed by reverse transcription-polymerase chain reaction (RT-PCR) and the early genes BARF1 and BHRF2 were also explored. The aim of this study was to establish the correlation between EBV-related genes and PCNA and c-myc gene expression and to identify the role of EBV-related genes in the pathophysiological process of EBVaGC.

## Materials and methods

### Subjects

The samples of gastric cancer and the pericancer tissues were obtained from inpatients who received surgical treatment in the Affiliated Hospital of Qingdao University Medical College, Qingdao Municipal Hospital and Yantai Yuhuangding Hospital, China, from January 2008 to December 2009. Of the 185 specimens, 13 EBV-positive cases were identified by PCR and southern blotting. Additionally, 45 cases that were paired with the 13 cases in age, gender, pathological type and clinical phase served as the controls. In the 58 cases, there were 48 males and 10 females, with an average age of 58 years (range, 31–81 years). All cases were confirmed by pathological examination. The study was approved by the ethics committee of Yantai Yuhuangding Hospital, Yantai, China. Informed consent was obtained from each patient.

### Reagents and materials

A reverse transcription kit (Promega Corporation, Madison, WI, USA), TRIzol (Gibco, Carlsbad, CA, USA), Hybond-N^+^ membrane (Amersham Pharmacia Biotech, Piscataway, NJ, USA), digoxigenin (DIG) oligonucleotide 3′-end labeling kit, CSPD, DNA molecular weight marker VIII (Roche Diagnostics, Mannheim, Germany), Taq polymerase and deoxyribonucleotide triphosphate (dNTP; Sangong Biotech, Shanghai, China) were used in this study. The monoclonal antibody for c-myc, 9E10, PCNA, PC10-vascular endothelial growth factor (VEGF) and SP-type immunohistochemistry kits were purchased from Zhongshan Golden Bridge Biotechnology Co., Ltd. (Beijing, China). All primers and probes were synthesized by Beijing Saibaisheng Biotech Co., Ltd. (Beijing, China).

### RNA extraction

The total RNA was extracted with TRIzol following the manufacturer’s instructions for the reverse transcription kit. cDNA was synthesized and served as the template for the PCR reaction. The specific primers and probes for the latent and the early phase gene were designed according to previous studies (4–7). All primers and probes are listed in Table I. The oligo probe was marked with the DIG oligonucleotide 3′-end labeling kit, following the manufacturer’s instructions.

### PCR

A 30 *μ*l PCR system was employed with 1.0 IU Taq DNA polymerase, 3 *μ*l 10X buffer, 1.5 mmol/l MgCl_2_, 0.1 mmol/l deoxyribonucleotide triphosphate (dNTP), 0.5 *μ*mmol/l each upper and lower primer and 3 *μ*l cDNA product. The PCR system was denatured at 94°C for 5 min, followed by 35 cycles with the following conditions: 45 sec at 94°C, 45 sec at 58°C, 1 min at 72°C and 10 min at 72°C. For glyceraldehyde 3-phosphate dehydrogenase (GAPDH), 25 cycles were completed.

The cDNA from the lymphoblastoid cell line (LCL) was employed as the negative control and the Ramos cell line was used as the positive control for EBV. The 10 *μ*l PCR product was detected by 2% agarose gel containing 0.5 *μ*g/ml ethidium bromide under ultraviolent radiation. The gel was transferred to a Hybond-N^+^ membrane and assessed by southern blotting.

### Immunohistochemistry (IHC) for c-myc and PCNA

The antibody was diluted 150-fold for c-myc, 200-fold for PCNA and 100-fold for VEGF. Phosphate-buffered saline (PBS) was employed as the negative control and a known breast carcinoma specimen was used for the positive control. All IHC experiments followed the manufacturer’s instructions.

The scoring criteria for immunohistochemistry were as follows: i) c-myc, the positive signal for c-myc existed in the nucleus as brown granular shapes. Five visual fields were randomly selected. The samples were regarded as positive when the positive area was >25% in the nucleus. ii) PCNA, the positive signal for PCNA existed in the nucleus as yellow particles. PCNA labeling index (PCNA LI) was calculated as the percentage of positive cells in 1,000 tumor cells.

### Statistical analysis

All data were analyzed with SPSS software (SPSS Inc., Chicago, IL, USA). The quantitative data were expressed as mean ± standard deviation (SD). The difference between the groups was compared with t-test or χ^2^ test. P<0.05 was considered to indicate a statistically significant difference.

## Results

### Expression of virus-related genes in the EBVaGC tissues

**Figure 1:**
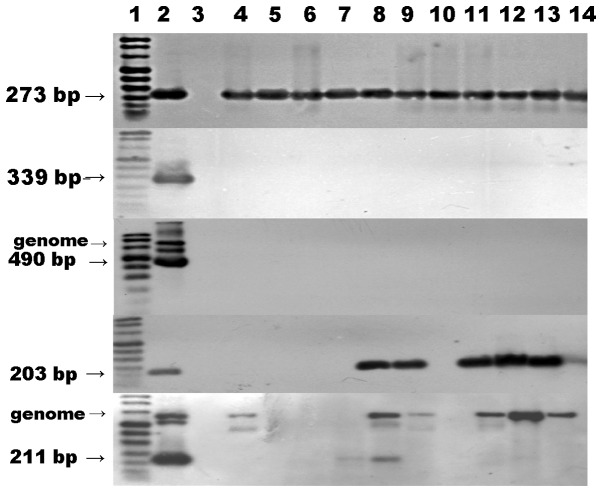
RT-PCR-southern blot analysis for EBNA1, EBNA2, LMP1, BARF1 and BHRF1 gene expression. Lane 1, DNA molecular weight marker VIII Digoxigenin-labeled; lane 2, LCL was used as a positive control for the detection of EBNA1; lane 3, Ramos cells (negative control); other lanes, EBVaGC specimens. RT-PCR, reverse transcription-polymerase chain reaction; EBNA, Epstein-Barr nuclear antigen; LMP1 latent membrane protein 1; LCL, lymphoblastoid cell line; EBVaGC, Epstein-Barr virus-associated gastric carcinoma.

The internal marker gene GAPDH was assayed in all 13 samples, which demonstrated the the success of PCR amplification. The expression of EBNA1 mRNA was identified in all cases; however, the expression of EBNA2 and LMP1 was negative in all cases. The expression of BARF1 was identified in 6 cases and BHRF1 in 2 cases. The RT-PCR and southern blotting results are shown in Fig. 1.

### PCNA immunohistochemistry

**Figure 2:**
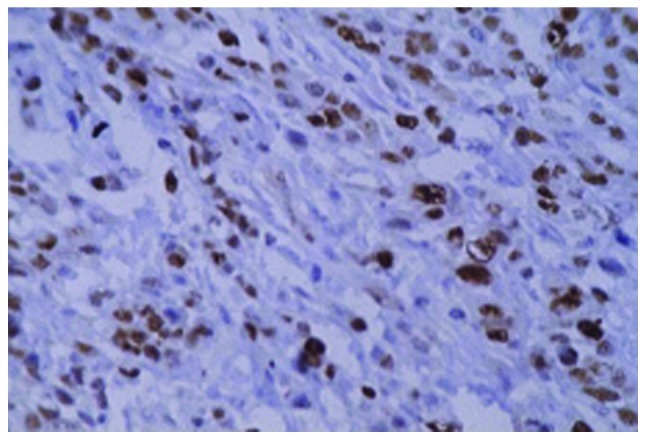
PCNA staining in EBVaGC. PCNA, proliferatng cell nuclear antigen; EBVaGC, Epstein-Barr virus-associated gastric carcinoma.

**Figure 3:**
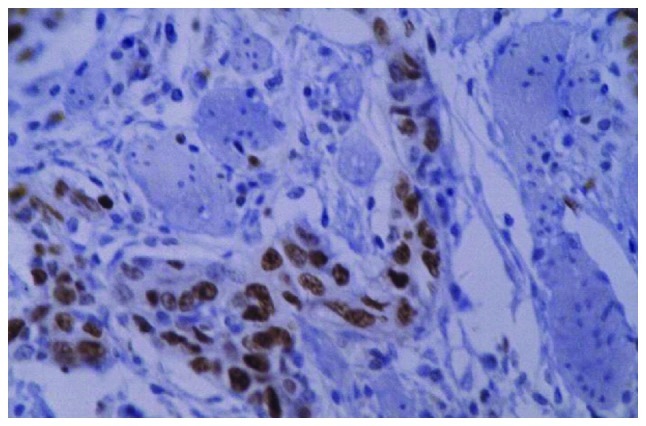
PCNA staining in EBVnGC. PCNA, proliferatng cell nuclear antigen; EBVnGC, Epstein-Barr virus-negative gastric carcinoma.

The positive signal was observed as brown granules in the nucleus (Figs. 2 and 3). The PCNA LI were 49.3768±12.1823, 14.8396±7.1847, 35.6139±8.3831 and 24.2735±10.1332 in the EBVaGC, the EBVaGC neighboring tissue, EBVnGC and the EBVnGC neighboring tissue, respectively. The difference between EBVaGC and EBVnGC was significant (t=4.686, P<0.01) and the difference between EBVaGC and the neighboring tissue was also significant (t=8.805, P<0.01). In all 58 cases, PCNA LI was significantly higher in the c-myc-positive group than in the c-myc-negative group (t=9.687, P<0.01). The results are listed in Table II.

### c-myc immunohistochemistry

**Figure 4:**
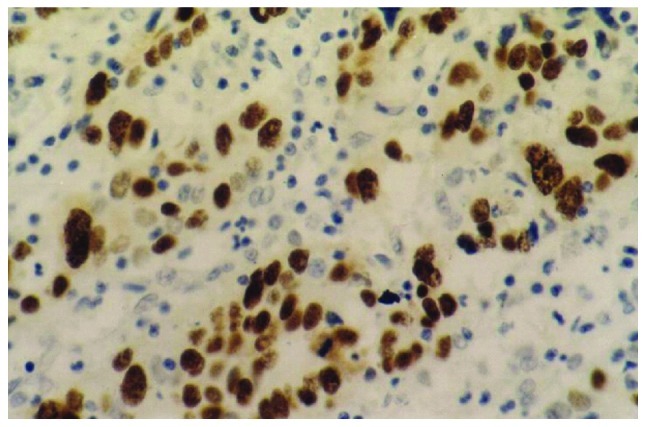
Overexpression of c-myc protein in gastric carcinoma tissue. Immunohistochemical detection of c-myc in paraffin section. Expression of c-myc was observed in nuclear of gastric carcinoma cells.

**Figure 5:**
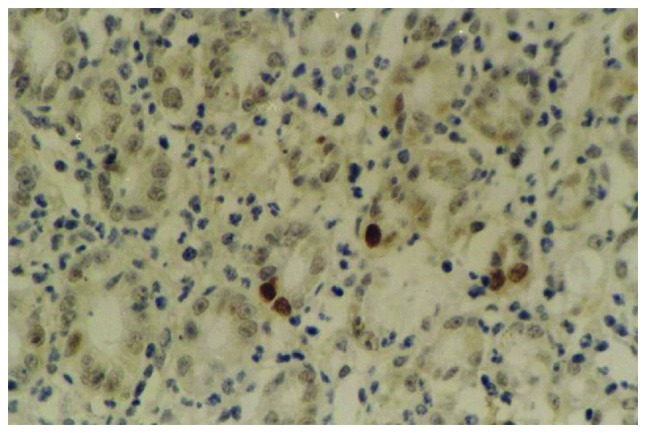
Low expression of c-myc protein in gastric carcinoma tissue. Immunohistochemical detection of c-myc in paraffin section. Expression of c-myc was observed in nuclear of gastric carcinoma cells.

**Figure 6:**
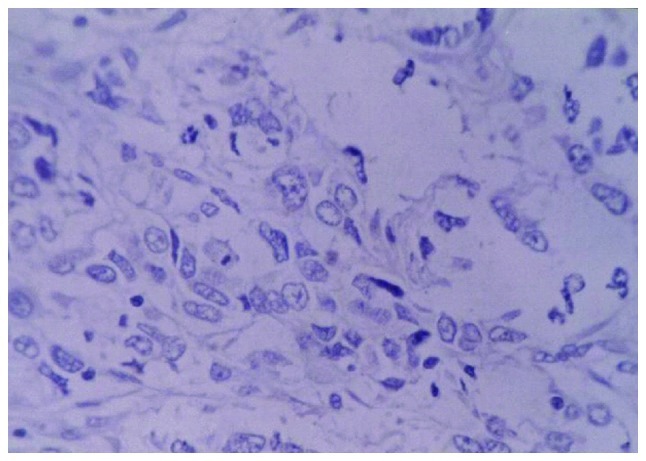
Negative c-myc protein in gastric carcinoma tissue. Immunohistochemical detection of c-myc in paraffin section. Expression of c-myc was not observed in nuclei of gastric carcinoma cells.

c-myc was observed in the cytoplasm, as shown in Figs. 4–6. The results demonstrated that the c-myc-positive rate in the 58 gastric cancer cases was 55.93% (33/58) and was 36.21% (21/58) in the neighboring tissues (χ^2^=4.989, P<0.05). The c-myc-positive rate in EBVaGC was 61.54% and in EBVnGC it was 55.56% (25/45). No significant difference was identified between these two groups (χ^2^=0.147, P>0.05). All results are listed in Tables III and IV.

## Discussion

In normal-dividing cells, the proliferation is partially regulated by c-myc, which plays an essential role in the regulation of cell proliferation and apoptosis. Besides, serving as a pro-oncogene, c-myc is closely related with tumor cell proliferation, transformation and the induction of apoptosis. c-myc is markedly increased in cells with fast division and low differentiation. Ishii *et al* (7) identified that in patients with EBVnGC, the expression of c-myc was significantly lower in the early phase than that in the proceeding phase. However, in EBVaGC patients, the level of c-myc is slightly higher in the early phase than that in the developing phase, although no significance was identified. It is considered that EBV affects the expression of c-myc. In the early phase of EBVaGC, EBV induces the expression of c-myc and inhibits the over-expression of p53, promoting cell proliferation. However, in the developing phase, EBV inhibits the expression of c-myc and the apoptosis of tumor cells, which makes therapy more difficult for late-phase gastric cancer. In our study, we did not identify a significant difference between the positive rate of c-myc in EBVaGC and EBVnGC. This suggests that there is no clear correlation between c-myc gene expression and the infection of EBV. The underlying reason may be due to sample bias. In our study, the samples were obtained from developing and later-stage patients. In these cases, the inducing effect of EBV on c-myc is lower than that in the early phase.

PCNA plays an important role in the initiation of cell proliferation. This molecule is observed in proliferating cells or cells that demonstrate a proliferating tendency. Therefore, PCNA reflects the proliferating condition of the whole cell population. In our study, the content of PCNA LI was significantly higher in EBVaGC cases than in EBVnGC cases, which suggests that there is a correlation between the expression of PCNA and EBV infection. EBV infection promotes the expression of PCNA. We also identified that the level of PCNA LI was clearly higher in the c-myc-positive group than that in the c-myc-negative group. This indicates that c-myc expression is closely related to the level of PCNA.

The viral proteins expressed in each phase during EBV replication have shown the potential effect on cell proliferation. A study has demonstrated that EBV-encoded LMP1 and BHRF1 are the major genes closely conneted with cell proliferation and c-myc levels. Yang *et al* (8) identified that EBV LMP1 induced the expression of endogenous c-myc. Despite this fact, it remains elusive whether LMP1 regulates the expression of c-myc directly or via the NF-κB pathway or other signal transduction pathways.

By analyzing 55 nasopharyngeal carcinoma specimens, Li and Zong (9) identified that the index of PCNA in the EBV LMP1-positive samples was significantly higher than that in the EBV LMP1-negative samples. This indicates that EBV LMP1 may promote the proliferation of tumor cells. Another study determined that EBNA2 promotes the expression of c-myc. In our study, neither LMP1 nor EBNA2 were expressed in the 13 samples, which indicates that cell proliferation and the expression of c-myc is independent of LMP1 and EBNA2.

The EBV early gene BARF-1 is regarded as the second main viral oncogene after LMP1. Studies have identified that BARF1 activates the expression of c-myc and B cell lymphoma 2 (Bcl-2) (10,11). zur Hausen *et al* (5) identified transcriptional BARF1 mRNA in 90% of the EBV-related gastriointestinal cancer cases. Despite the LMP1 mRNA-negative results, the BARF1 protein still exists in the epithelial cells. Therefore, BARF1, surrogating LMP1, plays a role in tumorigenesis. In our study, 6 EBVaGCs samples were BARF1-positive, which provides new evidence for its role in cancer. The correlation between the BARF1 gene, cell proliferation and c-myc gene expression requires further investigation with a greater number of cases.

BHRF1 and Bcl-2 share a highly homologous gene sequence. Similar to Bcl-2, BHRF1 inhibits cell apoptosis, promotes cell growth and transforms and elongates cell lifespan. Huang *et al* (12) observed a high-efficiency BHRF1-expressing clone in the CNE2 cell line and examined the biological activity following ^60^Co irradiaton. The authors identified that BHRF1 inhibits the expression of PCNA, ameliorates the sensitivity to the radiation and promotes the survival of tumor cells under nutrient-deficient conditions and contributes to the genesis and development of tumor cells. In our study, only two EBVaGCs samples demonstrated positive expression of BHRF1 and the other 11 cases demonstrated negative expresion. Combined with results from the study by Huang *et al*, we consider that BHRF1 inhibits cell proliferation and promotes the advancement of tumor cells in specific circumstances, including malnutrition, despite the fact that it does not contribute to cell proliferation in physiological conditions.

## Figures and Tables

**Table I:** Oligonucleotide primers and probes of EBV related genes used for RT-PCR analysis.

Transcripts	Primer sequences (5′-3′)	Product size (bp)
EBNA1		
5′ primer	GATGAGCGTTTGGGAGAGCTGATTCTGCA	273
3′ primer	TCCTCGTCCATGGTTATCAC	
Probe	AGACCTGGGAGCAGATTCAC	
EBNA2		
5′ primer	GCTGCTACGCATTAGAGACC	339
3′ primer	TCCTGGTAGGGATTCGAGGG	
Probe	CAGCACTGGCGTGTGACGTGGTGTAAGTT	
LMP1		
5′ primer	TCCTCCTCTTGGCGCTACTG	490
3′ primer	TCATCACTGTGTCGTTGTCC	
Probe	GAACAGCACAATTCCAAGGAACAATGCCTG	
BARF1		
5′ primer	GGCTGTCACCGCTTTCTTGG	203
3′ primer	AGGTGTTGGCACTTCTGTGG	
Probe	CTGGTTTAAACTGGGCCCAGGAGAGGAGCA	
BHRF1		
5′ primer	GTCAAGGTTTCGTCTGTGTG	211
3′ primer	TTCTCTTGCTGCTAGCTCCA	
Probe	ATGCACACGACTGTCCCGTATACAC	

EBV, Epstein-Barr virus; RT-PCR, reverse transcription-polymerase chain reaction; EBNA, Epstein-Barr nuclear antigen; LMP, latent membrane protein.

**Table II:** Correlation between c-myc expression and PCNA LI in 58 cases of gastric carcinoma.

	N	LI
c-myc (+)	33	51.645±9.172
c-myc (−)	25	32.013±4.916

Data are presented as mean ± standard deviation. t=9.687, P<0.01. PCNA LI, proliferating cell nuclear antigen labeling index.

**Table III:** Comparison of c-myc-positive rate between the gastric carcinoma tissues and corresponding paracarcinoma tissues.

Gastric carcinoma	Corresponding paracarcinoma tissue		Total
	c-myc (+)	c-myc (−)	
c-myc (+)	15	18	33
c-myc (−)	6	19	25

χ^2^=4.989, P<0.05.

**Table IV:** Comparison of c-myc protein expression between EBVaGC and EBVnGC.

	N	c-myc (+)	c-myc (−)
EBVaGC	13	8	5
EBVnGC	45	25	20

χ^2^=0.147, P>0.05. EBVaGC, Epstein-Barr virus-associated gastric carcinoma; EBVnGC, Epstein-Barr-negative gastric carcinoma.
